# Bone Health Deterioration in Transfemoral Prosthetic Users: An Analytical Biomechanical Explanation

**DOI:** 10.1002/cnm.70014

**Published:** 2025-02-03

**Authors:** Jose L. Zavaleta‐Ruiz, Matthew J. Major, Pankaj Pankaj

**Affiliations:** ^1^ School of Engineering The University of Edinburgh Edinburgh UK; ^2^ Department of Physical Medicine & Rehabilitation, Department of Biomedical Engineering Northwestern University, Jesse Brown VA Medical Center Chicago Illinois USA

**Keywords:** finite element analysis, ischial containment socket, mechanostat theory, osteopenia, transfemoral amputation

## Abstract

There is a five‐decade recorded history indicating that persons with transfemoral amputation experience bone loss in their amputated femur at levels seen in bedridden and post‐menopausal individuals, irrespective of age or mobility levels. We used computer simulation to recreate the mechanical environment created by the mechanical design of a prosthetic device in the surviving femur of individuals with transfemoral amputations. Finite element models of gait instances were developed from the hip joint computerized tomography scan of a subject along with a coupled ischial containment prosthetic socket fitted as per standard clinical guidelines. Accompanying mirror models, assembled similarly but without the prosthetic socket were used for stimulus comparison. Simulation showed that more than 90% of the trabecular bone volume in the amputated femur with an ischial containment socket registered compressive strain magnitudes below 300με. These strain magnitudes are below the threshold for bone maintenance as per mechanotransduction theory (i.e., they lie within the disuse window). Only 50% of the bone was in the disuse window for the mirror model for the gait instances considered. These results are consistent with reported in vivo evidence which shows that transfemoral prosthesis users may lose bone mass irrespective of age or mobility levels when using traditional socket designs. Clinically, this study shows that prosthetic sockets that support load through the ischium alter the kinetic chain and preclude application of mechanical stimulus that sustains healthy levels of bone mass in the proximal femur. The study also shows that femur length, prosthetic alignment and tissue tone influence this stimulus.

## Graphical abstract

Otherwise healthy individuals with transfemoral amputation have a five‐decade history of bone mineral loss in their residual limb, irrespective of age and mobility levels. Our computational analysis of the surviving femur under the effect of a prosthetic socket shows compressive and tensile stimulus magnitude between ±300μɛ, a threshold typically considered as the “disuse window” in the mechanotransduction theory. We show that the interaction of the pelvis with the ischial reliant prosthetic design reduces mechanical stimulus and promote demineralization.

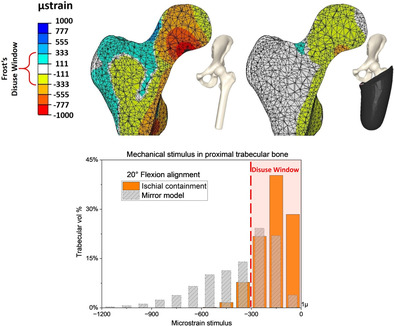

## 1 Introduction

The loss of bone mineral density (BMD) has been extensively reported in individuals with transfemoral amputation (TFA) [1, 2, 3, 4, 5]. As early as 1969, studies investigating cortical and trabecular bone density in the amputated limb of individuals with TFA, using either x‐ray, dual‐energy x‐ray absorptiometry (DEXA), magnetic resonance imaging (MRI) or computerized tomography (CT) imaging, have consistently shown a lower BMD [6] compared to the limbs of able‐bodied individuals and also when compared to the amputated limb of persons with major lower limb amputation at transtibial or knee disarticulation levels [1, 2, 3, 4, 7, 8, 9, 10] suggesting that the prosthetic treatment unique to TFA may be a possible cause of this disparity.

Mechanisms have been proposed to help explain the observed phenomenon of localized BMD loss in persons with TFA. Some studies have proposed that gait compensations related to walking with a transfemoral prosthesis may lead to BMD loss through mechanisms of disuse as the amputated limb does not experience similar load magnitudes, orientations, and frequency as intact limbs [11, 12, 13, 14], possibly exacerbated by less overall activity and sound limb reliance, and hence reduced limb loading [11, 15]. However, this explanation is challenged by the observation of similarly decreased BMD in elite athletes and younger servicemen with TFA who engage in high‐demand activity and would regularly subject their amputated limb to elevated loading [5, 9]. Other studies propose irremediable rapid bone loss suffered during prolonged periods of bedrest acutely following amputation surgery and during recovery of walking ability [2, 16, 17]. However, evidence from patients with long bedridden periods due to spinal cord injuries and from astronauts with BMD loss due to microgravity exposure suggests that these individuals are capable of recovering BMD after undergoing a comprehensive muscle stimulation and load‐bearing programs [18, 19, 20]. Consequently, while the proposed mechanisms may partially contribute to observed BMD loss in persons with TFA, they do not provide the full story.

According to the trajectorial theory as per Wolff's law [21], it is likely that changing biomechanics due to new loading paths and patterns could modify BMD rather than simply limb disuse. Moreover, as per mechanostat theory, there is a minimum effective strain (MES) that activates bone development [22]. MES under 300 με, a range termed the ‘disuse window’, can activate osteoclast activity that lowers BMD while ranges above 800 με (usually reached in able‐bodied mobility‐related activities of daily living) set the bone into the maintenance phase [23, 24]. The trajectory of surviving femur BMD loss in individuals with TFA would be consistent with this disuse window, but there is a need to reconcile the limitations of previous postulates for this loss and identify a potential mechanical source to explain the lack of BMD gains. As BMD loss in persons with TFA appears to occur irrespective of age and mobility level, one facilitating factor may be the mechanical environment enforced by the prosthetic socket that serves as a coupling and load transmission between the amputated limb and the prosthesis. Importantly, this mechanical environment is dependent on TFA socket design factors, including regions of rigid and flexible material, geometry, and suspension type. Although socket design is rarely reported in medical imaging studies on bone health in persons with TFA, the predominantly used socket design since the 1980s has been the ischial containment socket (ICS) [25]. The ICS is designed to utilize the pelvis ischial tuberosity as a “bony lock” to be the main source of load bearing as ground forces are transferred from the prosthesis to the surviving anatomy [26], with the intention of limiting the load experienced by the sensitive distal end of the amputated femur and hence minimizing risk of tissue trauma. While the ICS has been successful, remains one of the most commonly used designs and considered as the standard of care [27], transmitting weight bearing load from the prosthesis to the pelvis and thereby bypassing the amputated femur, we hypothesize that this kinetic chain interruption may misdirect bone mechanotransduction to enter the mechanostat disuse window.

We aim to create a theoretical framework to explain the well‐established evidence that individuals with TFA experience BMD loss in the amputated femur. Therefore, the purpose of this study was to use computational modelling to examine the altered force transmission path while donning an ICS, and the residual mechanical environment of the amputated femur. This study investigates the ICS purposed alterations of the skeletal loading and the effect of clinical factors related to prosthetic socket fit and form, such as surviving femur length, hip angle alignment, and soft‐tissue tone between the ischium and the socket, on load transmission. Results from this study may help fill a gap in knowledge on mechanisms related to BMD loss by quantifying strain stimulus levels in transfemoral prosthesis users and if any modifiable features may be tuned to take advantage of the ICS design while maximizing bone health.

## 2 Methods

Skeletal geometries were developed from CT scans, accessed on 20th May 2022, of the pelvis and proximal femur (i.e., hip joint) of a single healthy individual from a study conducted at the University of Utah, authors had an institutional review board approval and informed consent from the test subject [28]. Due to the open‐access data and retrospective nature of this study, no institutional review board or ethics committee approval for this particular study was required. Pelvis and femur geometries were extracted using Simpleware ScanIP (Synopsis, California) and imported into ABAQUS (Dassault Systèmes, France) for finite element analyses (FEA). To describe the topological complexity of the used geometries, a mesh with a ten‐node tetrahedral elements of approximately 2.25 mm average edge length were used to mesh skeletal geometries. Mesh convergence studies showed that the average minimum principal strain in the key regions of the femur only deviated by 8 με (6.8%) with a five‐fold increase in element numbers. We selected the hip joint free of contrast material used in the original study since it disrupted the extraction of the skeletal geometries and was deemed unusable. Pelvic bone was extracted as a solid homogenous and isotropic material with cortical bone properties (Young's modulus = 15 GPa, Poisson's ratio = 0.3) [29], while femoral bone was differentiated between trabecular (Young's modulus = 150 MPa, Poisson's ration = 0.3) [29] and cortical structures (Young's modulus = 15 GPa, Poisson's ratio = 0.3) and both assumed homogenous and isotropic.

**FIGURE 1:**
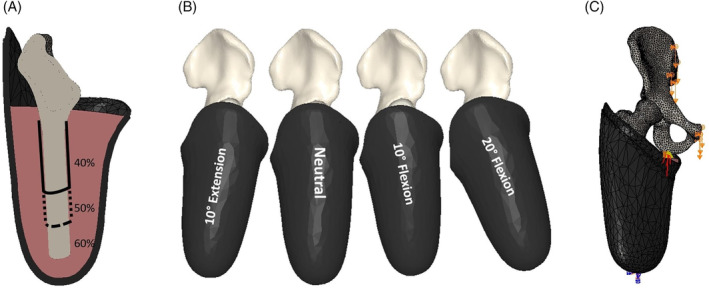
Extracted geometries from CT and 3D scans, assembled in ABAQUS. (A) Tested effect of surviving femur length at 40%, 50% and 60% of subject's expected length. (B) Surviving femur and socket‐tested sagittal alignment modifications. (C) Fixed boundary conditions were imposed at the apex of the socket and at the ischial seat, interaction between the socket and the ischial tuberosity is through four elastic connectors (in red), and loads are applied using displacement control from the pelvis (orange arrows).

Hip joint CT scans of the type used in this study often contain the skeletal structures encompassed between lumbar vertebrae and the proximal third of the femoral bone, capturing up to only a few millimetres distal to the lesser trochanter. In prosthetics practice a TFA at this level would be unusual and if required extremely complicated for prosthetic fitting due to such a short amputated limb. Therefore, we virtually contextualized limb heterogeneity by extending the femur to approximately 40%, 50% and 60% of its original length using the Carlyle formula [30], with the longest length maintaining an intact adductor attachment [31] (Figure 1A). We then imported 3D scans of an ICS and virtually generated perimeter soft‐tissue geometries that would serve as an exact match of the interior ICS profile (i.e., a positive geometry completely filling the negative space of the socket). While assembling the ICS model, we ensured that musculoskeletal landmarks were aligned with the socket following clinical prosthetic guidelines [26, 32]: (1) positioning the ischial tuberosity directly superior to the socket ischial ‘seat’, (2) cupping the greater trochanter by the socket lateral wall and (3) positioning the adductor longus relief in the medial anterior corner. The socket was characterized as a carbon fibre composite with Young's modulus of 1.5 GPa and Poisson's ratio of 0.3 [29].

Initial upright standing was simulated by positioning the two horizontal lines connecting the pelvis anterior inferior iliac spines and anterior superior iliac spines horizontal lines in parallel. Then, using the greater trochanter as an axis, we rotated the femur in the sagittal plane to recreate gait sagittal hip motion experienced during early, mid, and terminal stance (i.e., 20° of hip flexion, 10° of hip flexion, 0° or neutral, and 10° of hip extension) (Figure 1B). In all cases, the femur transverse position as extracted from the CT image was retained, and the hip joint *Q* (adduction) angle was 10° as clinically recommended.

Restrained boundary conditions were imposed on the socket at the apex and below the ischial seat, the former representing socket attachment to the distal prosthesis and the latter representing the ‘bony lock’. This bony lock position was obtained following standard trochanter‐knee‐ankle line alignment principles [33]. Loads experienced during gait were applied using displacement control at four nodes distributed on each of the pelvis ilium (at sacrum union) and pubic symphysis, to result in equivalent applied forces as percentages of body weight (BW)—from 100% to 225%, in 25% increments, for a 100 kg individual (Figure 1C). We simulated an ideal prosthetic suspension that mated the skin to the socket interior wall through a node‐to‐node tie condition between soft‐tissue and socket geometries. Similarly, the femur's cortical surface was tied to both the soft tissue and trabecular surface. The socket‐pelvis interaction was simulated following established CAT‐CAM principles [26], modelled through four elastic connectors of 1.5 cm length with a parallel arrangement effective stiffness of 0.2 MPa and a maximum displacement of 5, 2.5 and 0 mm [34] to represent three model cases of tissue (gluteus muscle and skin) thickness compressibility, hereafter designated as SOFT, MEDIUM and FIRM, respectively.

Accompanying mirror models, which did not have the socket, were subjected to the same loads as cases with the ICS. These mirror models had the same geometries, material properties and boundary conditions, as the model with ICS. These models served as the baseline comparison for TFA simulations.

Our primary outcome from the FEA simulations was evaluating varying strain levels in the femoral bone for the range of cases and in each case computing volumes that fell in the disuse window [19, 22, 23, 24].

## 3 Results

**FIGURE 2:**
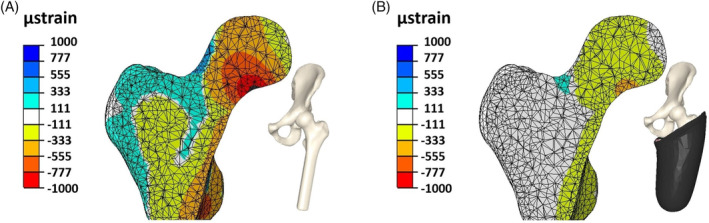
Computed micro‐strain contours in proximal trabecular bone as a result of body weight of 225% at hip neutral alignment due to kinetic chain (A) without socket influence and (B) for an ischial containment user. The plot shows both max compressive (negative) and max tensile (positive) principal strains.

Typical minimum principal (compressive) strain contours for a representative person with TFA (neutral alignment, 60% amputated limb length, 5 mm soft tissue between ischial tuberosity and the socket) and for the mirror model comparison subjected to 225% BW loading are shown in Figure 2. The strain contour pattern variation for the mirror model (Figure 2A) compares well with a previous open‐access study [35] that employed an extremely detailed model, providing verification of our computational model. Figure 2B demonstrates that the addition of the ICS reduces the strain magnitude and substantially reduces stimulus to the proximal femur.

**FIGURE 3:**
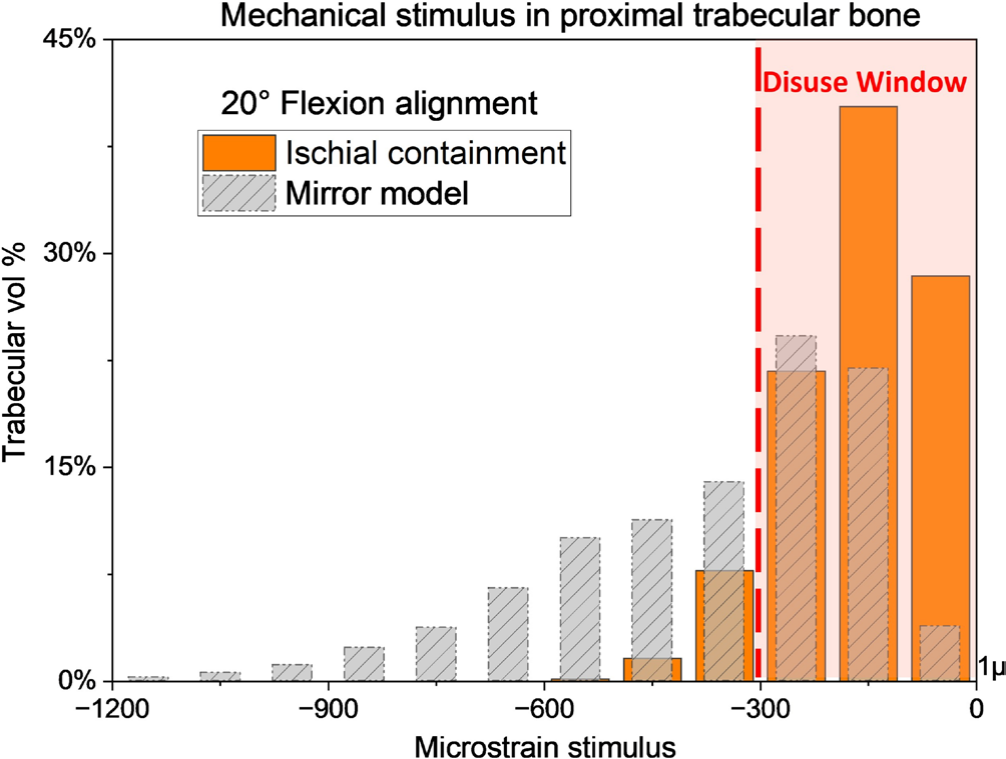
Mechanical loading stimulus of hip at 20° of flexion and compressed by 225% body weight with surviving femur length of 60%. Histogram compares stimulus registered in proximal trabecular bone in able‐bodied (grey) and under the influence of an ischial containment socket with 5 mm of compression allowance—soft‐tissue tone (orange).

Figure 3 compares the volume of minimum principal strain values for a representative TFA model (soft tone with 20° hip flexion) and the mirror model loaded at 225% BW, showing that only 7.5% of trabecular bone remains outside the disuse window for the TFA model compared to 50% in the mirror model.

**FIGURE 4:**
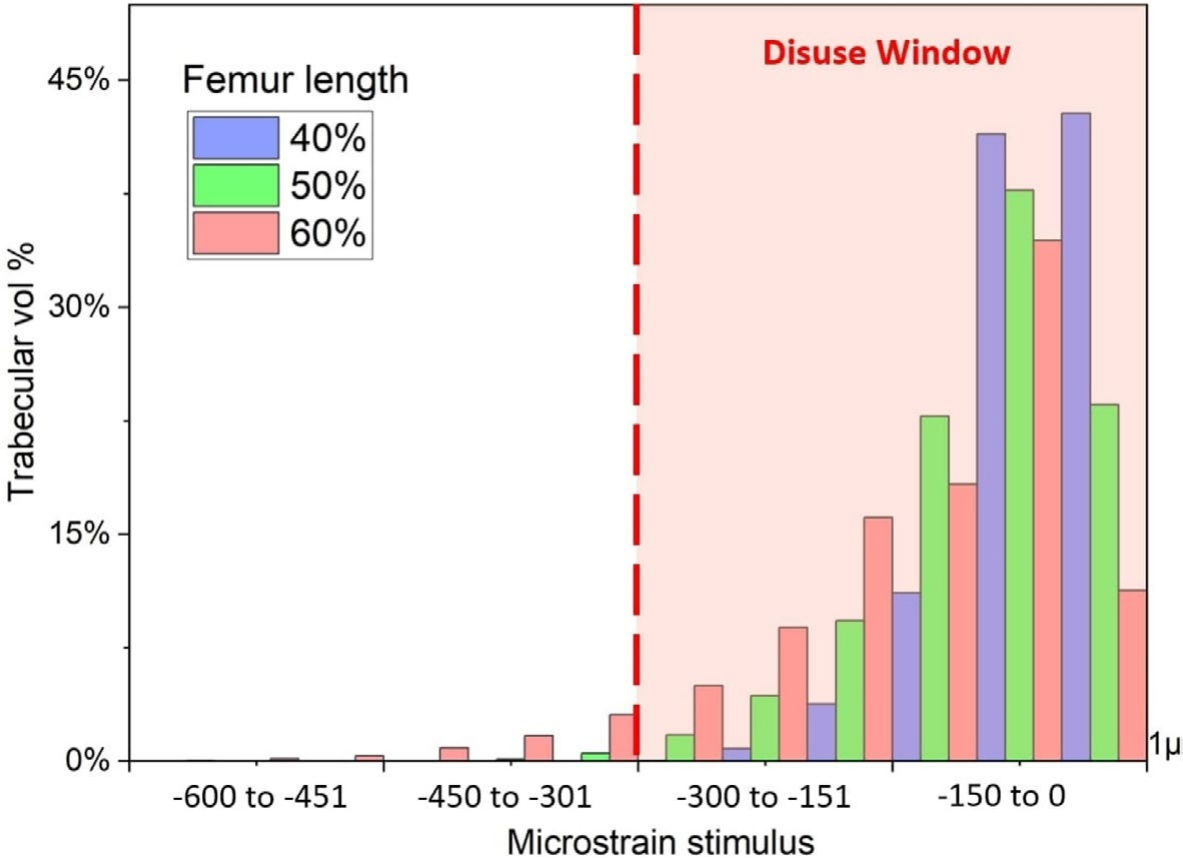
Stimulus registered in proximal trabecular bone volume for surviving femur length of: 60% (red), 50% (green) and 40% (blue), with socket at 20° of flexion sagittal alignment, under 225% body weight and tissue compressive allowance of 5 mm.

Figure 4 compares the volume of minimum principal strain attained at 225% BW for TFA models with different amputated limb lengths. Other parameters kept constant are neutral alignment and 5 mm soft tissue between the ischial tuberosity and the socket. We observed a positive relationship between amputated limb length and the volume of bone experiencing compressive strains above the disuse window. For femur extensions of 40% and 50% of the original limb length, more than 99.5% of the trabecular bone stimulus was below 300με magnitude, while this percentage decreased to 98.5% for a length of 60%.

**FIGURE 5:**
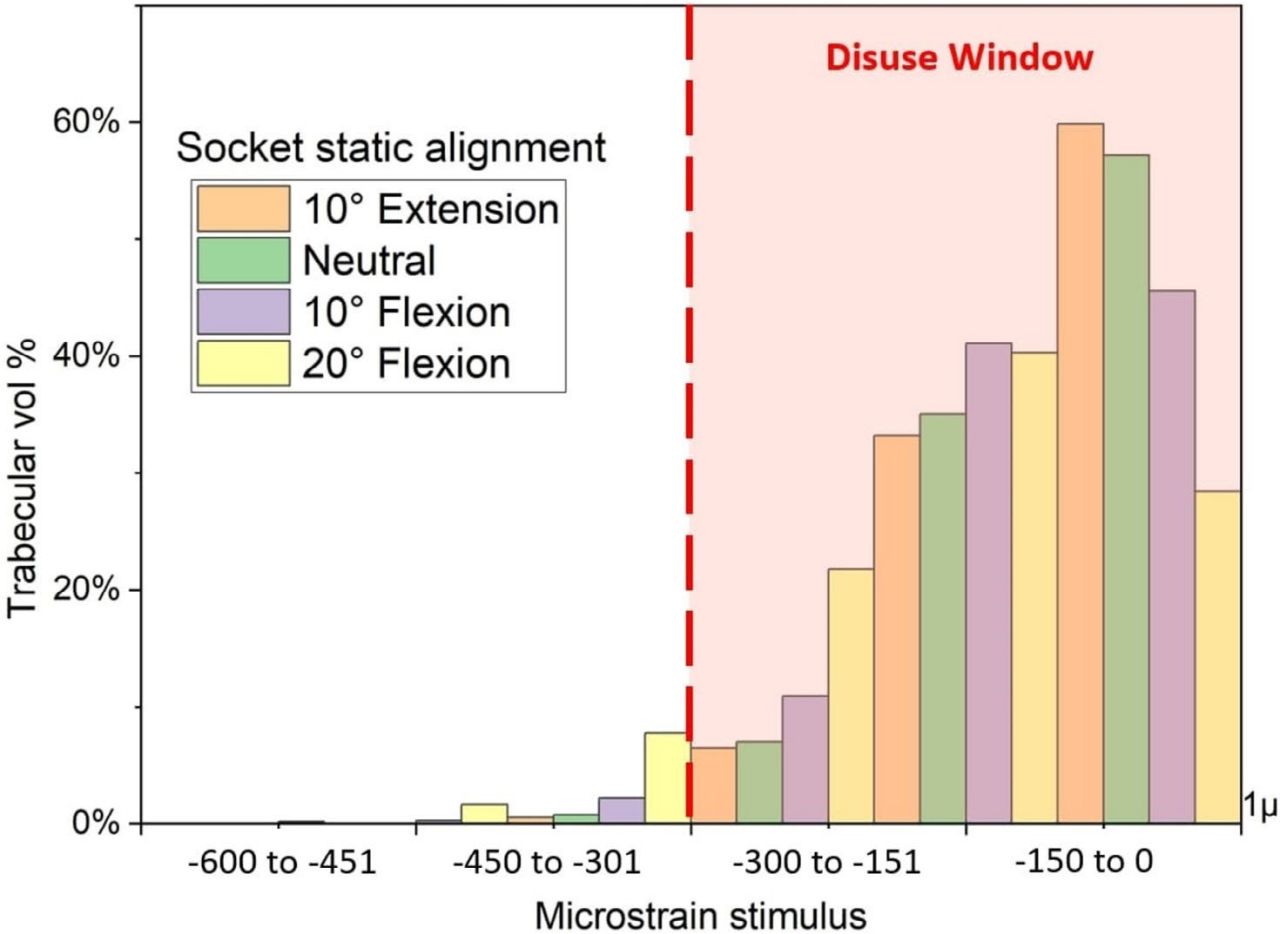
Mechanical loading stimulus registered in proximal trabecular bone by socket alignment, for model with surviving femur length at 60%, 5 mm tissue tone compressibility allowance and 225% body weight force input. Flexion alignments (purple and yellow bars) show higher stimulus than neutral (green) and extension (orange) alignments.

Figure 5 demonstrates the effect of socket alignment with femur at 60% length. Extension and neutral alignment at 225% BW loading registered 99% stimulation inside the disuse window, while with 10° and 20° flexion, volume inside the window was 97.3% and 92.5%, respectively.

**FIGURE 6:**
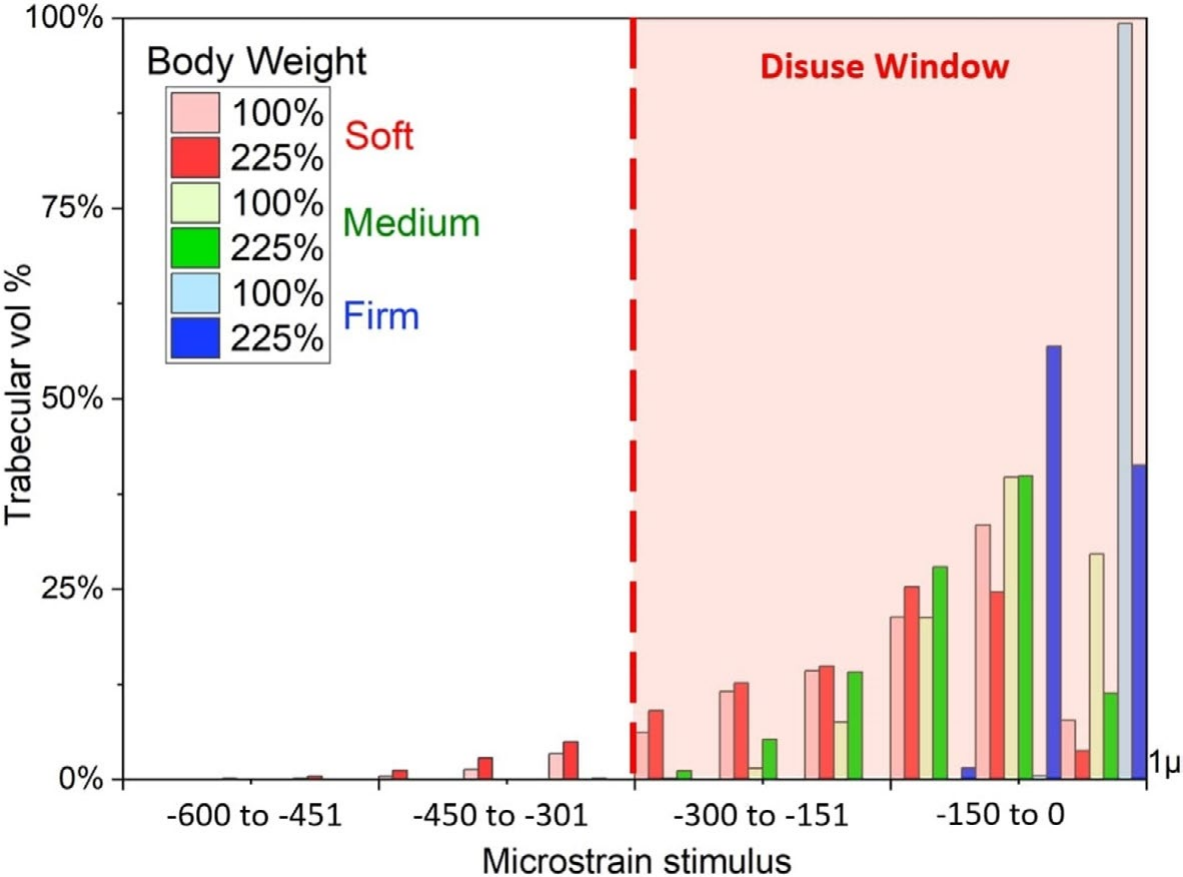
Stimulus registered in the proximal trabecular bone volume due to the effect of soft‐tissue tone: Soft (red), medium (green) and firm (blue), with femoral length at 60%, under 225% body weight magnitude and 20° of hip flexion.

The simulation results suggest a positive relationship between greater ischium soft‐tissue compression and trabecular compressive strain stimulus. Results indicate that for firm and medium tone, all bone remains inside the disuse window, while soft tone allows 1.6% of trabecular bone to escape the disuse window. Figure 6 displays the stimulus magnitude for the soft tone model at 20° flexion, which increased the volume of trabecular bone stimulated outside the disuse window to 7.5%.

Overall, our results indicate that the addition of an ICS modifies the socket‐pelvis load path and reduces femur strain stimulus below the MES. We computed the percentage of total force being transmitted to the ischium via the socket under varying loads (Table 1), and two in vivo recordings of pressure experience in the socket seat were used for independent model validation [36, 37]. Tissue tone influences the force transmission, with firm and soft tones transferring the largest and smallest percentage of forces, respectively, irrespective of socket alignment. Moreover, there was little change in the proportion of forces being transmitted via the ischium with increased load application for firm tone while the proportion increased substantially for soft tone and moderately for medium tissue tone.

**TABLE 1:** Resultant force variation concentrated at the ischial tuberosity, in the ischial containment model assembled with 60% femoral length.

		Body weight	% force experienced in the ischium‐socket region			
			10° extension	Neutral	10° flexion	20° flexion
Soft‐tissue tone	Firm	100%	79%	68%	67%	71%
		125%	79%	68%	67%	71%
		150%	79%	68%	67%	71%
		175%	79%	68%	67%	71%
		200%	79%	68%	67%	71%
		225%	79%	68%	67%	71%
	Medium	100%	55%	47%	42%	55%
		125%	61%	51%	46%	59%
		150%	64%	54%	50%	61%
		175%	67%	56%	53%	63%
		200%	68%	58%	54%	64%
		225%	69%	59%	55%	65%
	Soft	100%	15%	23%	14%	23%
		125%	35%	33%	26%	39%
		150%	45%	39%	31%	46%
		175%	52%	44%	38%	51%
		200%	55%	47%	41%	54%
		225%	58%	49%	44%	56%

## 4 Discussion

The primary aim of this study was to evaluate the mechanical strain stimulus experienced by the surviving femur in individuals wearing an ICS, which has established ramifications on bone health [38, 39]. Overall, results from this study suggest that ICS's ‘ischial seat’ absorbs up to 79% of BW (hip position dependent), as clinically intended [26, 32] (Table 1), which provides an element of validation for our models. Consequently, the force transmitted via the femur does not provide adequate stimulation to facilitate bone remodelling as per mechanostat theory [22, 23], a pattern sustained across all studied hip ranges of motions and BW loadings. The mechanical isolation effect caused by the ICS is seen in the 42% difference of trabecular bone stimulated above the disuse window between TFA and mirror models. This difference clearly indicates that the presence of the ICS significantly reduces the mechanical stimulus experienced by the amputated limb.

### 4.1 Mechanical Compression Effect of Prosthetic Practice Variables

While a linear relationship exists between load increments and strain in the mirror (without socket effect) model (if materials are assumed to be linear elastic), in the ICS model, strain response can be modulated by a number of factors. Findings suggest that longer surviving femurs, and hence increased lever arm and resulting forces, can generate higher compressive strains in the proximal femur. Even with loading at 225% BW, over 99% of trabecular bone remains within the disuse window for femoral extensions at 40% and 50% of the original CT length, while with a 60% length, the stimulus rising above the disuse window threshold varied from 6.1% to 8.1% depending on the hip angle (Figure 4) and the nonlinear soft‐tissue tone effect (Figure 6).

In this study it was found that increased soft‐tissue compressibility promotes a more natural kinetic chain interaction. The percentage force experienced at the ischium‐socket region is dependent on soft‐tissue tone. In the ‘soft’ case the tissue at the ischial tuberosity starts taking increasing load percentage as it gets compressed. This increase in load percentage reduces when the tissue starts to reach its maximum compression at higher load increments. For the ‘firm’ case, the ischial seat carries a large proportion of the load in all positions and for all load increments. Another interpretation of this result relates to the intimate capture of the ischial tuberosity by the prosthetist in socket fabrication, suggesting that pelvis targeted accuracy during amputated limb impression promotes a higher absorption of BW at the ‘ischial seat’, with a negative correlation to trabecular mechanical stimulus. The socket acts as an unyielding mechanical stop that modifies the natural kinetic chain. Hence, sockets that support load through the ischium hinder positive bone health by preventing remodelling from occurring and this is found to happen in all hip position scenarios considered in this study.

### 4.2 Does the Prosthetic Mechanical Environment Align With Expected Disuse Osteopenia According to the Mechanotransduction Theory

Our results suggest that the design intention of an ICS, namely to encourage load transmission through the ischium rather than the surviving femur, appears to create a mechanically strain‐impoverished environment for the underlying bone tissues (Figure 2B) [26, 32]. While the socket is designed to minimize the risk of amputated limb trauma brought on through repetitive strain injury, its rerouting mechanism results in partial mechanical isolation of the femur leaving it in a largely ‘floating’ state inside the socket. Isolating the amputated femur from the kinetic chain, a phenomenon we term the *floating femur*, may be a primary contributor to BMD loss seen in individuals with TFA who regularly use a clinically optimized ICS [25]. Importantly, our study hypothesis could contextualize the differentiated systemic osteoporosis diagnosis from the *localized unloading osteopenia* diagnosis in individuals with TFA as suggested by McMenemy et al. [39].

### 4.3 Limitations

The presented models have been reconstructed using retrospective and open‐source data. The CT‐extracted skeletal geometries were not from an individual with amputation and hence the imported socket geometry was not clinically designed, overlooking the hydrostatic pressure relation between the outer perimeter of the soft tissue and internal socket walls. However, given that this pressure has been previously measured to be below 30 kPa it is unlikely it will alter the bone's compression response. This setup was sufficient to achieve our aim of an in silico recreation of the loading trend of a surviving femur donning an ICS design and the subsequent mechanostransduction effects.

The models do not explore bone molecular mechanotransduction and ignore the mechanical contributions of muscle contraction, patent in the lack of stimulation seen in Figure 2B at the greater trochanter where there is insertion of the gluteus medius. Despite evidence showing that muscle force‐induced bone stress influences bone remodelling [40], following musculature loss of attachment points and an estimated force loss of over 70% [41], the in silico representation of the prosthetic socket and the skeletal bodies' reaction to load bearing, as produced in this study offers insight into the mechanical environment of using an ICS and a possible mechanism for BMD loss in transfemoral prosthesis users.

Some previous investigations have used equivalent strain (ES) and strain energy density (SED) to predict BMD remodelling distribution [42, 43]. However, this study assumes that minimum principal (compressive) strain is a valid indicator of bone mechano‐regulation, aligning with the mechanostat theory [22, 23]. It is unlikely that use of SE and SED measures would alter the conclusions of the study. Lastly, these results are based on a simulation, which requires in vivo validation. As the strain environment within the femur cannot be directly measured, one future validation approach could involve a comparison of predicted joint reaction forces via subject‐specific FEA simulation and those estimated from matched in vivo data via inverse dynamics.

## 5 Conclusion

A number of studies have attempted to describe the rationale behind BMD loss in individuals with a TFA. Our FEA computational study suggests that the mechanical design of an ICS might be partially responsible as it reroutes loads from the prosthesis to the pelvis, thereby allowing the bone tissue strain in the femur to remain within the ‘disuse window’ per mechanostat theory. However, it seems that tuning the socket's ischial ‘seat’ distance to the ischial tuberosity could alter the strain landscape and move trabecular bone out of the disuse window. Future work needs to investigate if BMD levels in individuals with TFA can be recovered via traditional prosthetic treatment, without compromising comfort and soft‐tissue health, with a modified ‘ischial seat’ reliance that addresses this *floating femur* state.

## Author Contributions

J.L.Z.R., M.J.M. and P.P. conceived the presented idea and developed the theory, at their corresponding institutions. J.L.Z.R. performed the computations at The University of Edinburgh. P.P. verified the analytical methods at The University of Edinburgh. M.J.M. provided clinical biomechanics expertise at Northwestern University. All authors discussed the results and contributed to the final manuscript.

## Disclosure

Each author certifies that there are no funding or commercial associations (consultancies, stock ownership, equity interest, patent/licensing arrangements, etc.) that might pose a conflict of interest in connection with the submitted article related to the author or any immediate family members.

## Ethics Statement

Study participants were not involved in the design, conduct, interpretation, or translation of the current research. Data from cited study DOI 10.1002/jor.22040 (http://mrl.sci.utah.edu/software/hip‐image‐data) has been made available by the authors and had an institutional review board approval and informed consent from test subjects.

## Conflicts of Interest

The authors declare no conflicts of interest.

## Data Availability

The data that support the findings of this study are available from the corresponding author upon reasonable request.
